# Supplementary material for: Do young dating app users and non-users differ in mating orientations?
Source: PLoS One. 2021 Feb 2;16(2):e0246350. doi: 10.1371/journal.pone.0246350 (PMC7853474; doi:10.1371/journal.pone.0246350)
Supplement: S1 Appendix — (PDF) [file pone.0246350.s001.pdf]

S1 Appendix

Long Term Mating Orientation Scale [1] – Spanish Version

Indica tu grado de acuerdo o desacuerdo con cada una de las siguientes afirmaciones, en base a la siguiente escala de respuesta:

1 = Totalmente en desacuerdo 2 = En desacuerdo 3 = Moderadamente en desacuerdo 4 = Ni de acuerdo ni en desacuerdo 5 = Moderadamente de acuerdo 6 = De acuerdo 7 = Totalmente de acuerdo

1. Me interesa tener una relación romántica a largo plazo con alguien especial.	1	2	3	4	5	6	7
2. Espero tener una relación romántica que dure el resto de mi vida.	1	2	3	4	5	6	7
3. Me gustaría tener una relación romántica que dure para siempre.	1	2	3	4	5	6	7
4. Las relaciones románticas a largo plazo no son para mí.	1	2	3	4	5	6	7
5. Encontrar una pareja para una relación romántica a largo plazo no es importante para mí.	1	2	3	4	5	6	7
6. Puedo imaginarme fácilmente empezando una relación romántica a largo plazo con alguien especial.	1	2	3	4	5	6	7
7. Puedo imaginarme sentando la cabeza con alguien especial.	1	2	3	4	5	6	7

1. Jackson JJ, Kirkpatrick LA. The structure and measurement of human mating strategies: toward a multidimensional model of sociosexuality. Evol Hum Behav. 2007;28: 382–391. doi:10.1016/j.evolhumbehav.2007.04.005
