# Supplementary material for: Do young dating app users and non-users differ in mating orientations?
Source: PLoS One. 2021 Feb 2;16(2):e0246350. doi: 10.1371/journal.pone.0246350 (PMC7853474; doi:10.1371/journal.pone.0246350)
Supplement: Attachment — Submitted filename: Response_to_reviewers.docx [file pone.0246350.s003.docx]

**PONE-D-20-29867R1**

**Do young dating app users and non-users differ in mating orientations?**

**PLOS ONE**

First of all, we would like to thank the editor and the reviewer 3 for the time and effort invested in reviewing this manuscript. Your comments have been very useful to improve our work.

**COMMENTS BY REVIEWER #3**

**This is an interesting study conducted on a large sample assessing dating app use and mating orientations. Of particular interest is that the authors find no correlation between dating app use and LTMO. The authors have demonstrated a good attempt to address most of the reviewers feedback. However, I do have some recommendations for minor revisions.**

Response: We would like to thank Reviewer 3 for the time and effort invested in reviewing our manuscript and for her comments. Your suggestions have been very useful to improve our work.

**The analysis is appropriate to answer the research question - however, what was the rationale for not including STMO? Inclusion of this measure would have provided additional, interesting information (e.g., as the author themselves notes, that STMO and LTMO do not exist independently).**

Response: In this point we are unsure about what Reviewer #3 meant by STMO. We will distinguish between two potential meanings:

*1) STMO as the subscale from the questionnaire developed by Jackson and Kirkpatrick (2007).*

Among the reasons why Jackson and Kirkpatrick (2007) developed their questionnaire we can highlight two. First, because they wanted to clearly separate between short-term and long-term mating, as previous literature has tended to consider both constructs as opposite extremes from the same dimension. They considered and showed that it was not the case. Second, because the original SOI questionnaire (Simpson & Gangestad, 1991) mixed sociosexual attitudes (attitudes in favor of casual sex) and sociosexual behaviors (number of previous sexual partners). They proposed STMO items, which included three items from the SOI– tapped attitudes/comfort with casual sex and avoided the multidimensionality of the SOI, but it did not assess behaviors.

Importantly, Penke and Asendorpf (2008) presented, shortly after Jackson and Kirkpatrick's (2007) article was published, an improved SOI version, the SOI-R. In this improved version, the problems of multidimensionality of the SOI are also considered, but, instead of reducing the short-term orientation to attitudes, the SOI-R dimensions of behavior and desire were also included. So, the SOI-R questionnaire –the one used in this study– covers the same as the STMO plus two additional aspects of short-term orientation. Previous research has shown the advantages of considering these three interrelated but different aspects (e.g., Barrada et al., 2018). As all the SOI-R dimensions have been shown to present adequate psychometric properties and its content is wider than both the original SOI and the STMO, we found no reason to include STMO.

*2) STMO as the construct of short-term mating orientation.*

We included a measure of short-term mating orientation: the SOI-R. Although we acknowledge that the terms of 'sociosexuality' and 'short-term orientation' have not been always clearly defined, we consider that there are several reasons to understand unrestricted sociosexuality as equivalent to short-term orientation (e.g., Buss & Schmitt, 2019; Penke & Asendorpf, 2008). In the Introduction section we clearly noted that we would use both terms as interchangeable:

*Some studies, like those of Botnen et al. [8] and Grøntvedt et al. [19] have found that people with unrestricted sociosexuality (i.e., short-mating orientation) report to use these apps more.*

Also, in the Discussion we had written:

*Thirdly, among the contributions of the article should be highlighted the assessment of sociosexuality from a multidimensional point of view, distinguishing between behavior, attitudes, and desire, following the recommendations of other authors [15,38]. It has been shown that the three dimensions of the construct, understood as short-term orientation,*

The SOI-R measures sociosexuality, that is, it measures short-term orientation. As indicative of this, as we have said, the STMO questionnaire and the SOI share several items. One of the advantages of the SOI-R is that it clearly differentiates behavior, attitudes, and desire. Some of the research about short-term orientation have mixed these different dimensions.

In the previous version of the manuscript we discussed the differences between short- and long-term mating orientations in this way:

*This traditional view of mating orientation [short-term versus long-term] has been criticized by some authors, such as Jackson and Kirkpatrick [24], who claimed that short-term and long-term orientation are not two opposing poles in a single dimension, but two dimensions that, while negatively related, can be and should be differentiated. Thus, for example, it is possible to desire or be involved into a stable relationship and maintain multiple sexual relationships without commitment [27,28]. It is also possible to have no interest in any kind of relationship.*

*The conception of sociosexuality has also be refined. Different researchers have shown the appropriateness of abandoning the classic unidimensional stance of short-term orientations [18] and paying attention to a multidimensional perspective [15]. This more fine-grained approach includes sociosexual behavior (i.e., past sociosexual behavior), attitudes (i.e., positive appraisal about casual sex), and desire (i.e., sexual arousal with people with whom no committed romantic relationship exists).*

We have included a new paragraph to clarify some possible ambiguity in the terminology (p. 5, lines 96-108):

*However, it is still common that researchers continue to study mating strategies like opposing poles and sociosexuality from a unidimensional approach when they analyze demographic and psychological correlates. There is still some theoretical confusion in the use of some terms. For instance, Penke [29] defined restricted sociosexuality as the "tendency to have sex exclusively in emotionally close and committed relationships" and unrestricted sociosexuality as the "tendency for sexual relationships with low commitment and investment" (p. 622). This conceptualization assumes that (a) restricted and unrestricted sociosexuality define a single dimension and (b) that restricted is equivalent to long-term mating orientation and unrestricted to short-term orientation. While we agree with the first assumption, we have justified that short- and long-term mating orientation are not the two extremes of a single dimension. While unrestricted sociosexuality can be understood as interchangeable with short-term orientation, restricted sociosexuality is not long-term, but lack of short-term orientation.*

**Although I appreciate sociosexual might share variance with STMO, my understanding is: Restricted sociosexuality (i.e., preference for sex within long-term and committed relationships) and unrestricted sociosexuality (i.e., preference for short-term and no-strings-attached sex). Thus, we could argue that sociosexuality will also share variance with LTMO. Given the low(ish) correlations between sociosexuality and LTMO in Table 1, clearly they share variance but are still distinct. Thus, STMO could also have been included in addition to SOI-R.**

Response: As we have mentioned, from our point of view, it is not that sociosexual orientation shares variance with STMO, but that both are equivalent. As we have noted, both of their prototypical measures share several items. One of the reasons to use the SOI-R is that it clearly differentiates between several aspects of short-term (sociosexual) orientation, which implies that this measure offers richer information.

We have to disagree with the way how the Reviewer has characterized restricted sociosexuality ("preference for sex within a long-term and committed relationship"). By understanding sociosexuality in this way we would be considering mating orientations with a unidimensional approach, where long-term and short-term are opposite poles. As Jackson and Kirkpatrick (2007) argued and showed, and as our own results indicate, both orientations are negatively correlated, but cannot be considered as lying into a single dimension. If that was the case, our research could not offer anything new, as it has already repeatedly found that dating apps users show higher short-term orientation (e.g., Barrada & Castro, 2020). So restricted sociosexuality would be better understood as a lack of preference for short-term sex.

We agree with the idea that sociosexuality and LTMO share variance but are still distinct. We developed this idea in the Introduction.

As we have argued previously, the STMO questionnaire is partially based on the SOI (and the SOI-R on the SOI), so we find no reason to include the STMO. Also, we consider both constructs, short-term orientation and sociosexuality, as equivalent.

**The inclusion of a measure of STMO could have added richness to results. Apps used and SOI-B behaviour have a particular high correlation (Table 1). It is interesting that LTMO is not correlated to app use, but they are sociosexually unrestricted in their behaviour. All other SOI scales are correlated quite highly too. Theoretical implications of this suggest to me that perhaps it is time researchers step away from the conceptualisation of unrestricted = STMO, restricted = LTMO. People might be looking for a long-term partner, but also have an unrestricted sociosexuality.**

Response: As we have said, as short-term mating orientation and sociosexual orientation are interchangeable terms, we consider that nothing could be gained by including a (second) STMO measure, a redundant measure.

We agree with the idea that the conceptualization of unrestricted = STMO and restricted = LTMO is, although intuitive, wrong. That was the theoretical contribution from Jackson and Kirkpatrick (2007), a main source for our manuscript. It is not difficult to imagine people with no interest in neither short-term nor long-term or with interest in both of them.

We have included this in the Discussion section (p. 15, lines 298-302):

*This points to the need to step away from the conceptualization of unrestricted sociosexuality as equal to short-term mating orientation and restricted sociosexuality as equal to long-term mating orientation [29]. As we previously noted, restricted sociosexuality is better understood as lack of short-term orientation, what is not equivalent to long-term orientation.*

**In sum, if the authors did not include the STMO in a larger data set and it cannot be included, I think a discussion of why SOI is included and not STMO is required.**

Response: As we have said, we have theoretical reasons to consider unrestricted sociosexuality and short-term mating orientation as equivalent. For that reason, we find no reason to discuss why short-term orientation was not included as, in fact, it was.

**It is not a problem to have sampled young adults (or adopting Arnett categorisation, emerging adults); however, the rationale for this sample needs to be stronger. The choice for this age range needs to be embedded in the introduction discussion of orientations. Why, in particular, are you interested in young adults? Does their app use appear to be different? Their mating orientations? Given the evolutionary perspective applied, it could be particularly important to provide a rationale for assessing orientations of emerging adults (e.g., fertility?).**

Response: Thank you very much for giving us the opportunity to clarify this point. Our interest in this population of emerging adults is threefold. First of all, due to methodological reasons. As it appears in the previous version of the manuscript and we explain to one of the previous reviewers, we focus on this population (university students aged 18 to 26) to be consistent and coherent with the inclusion criteria of previous studies conducted in our research group (see, for example, Barrada et al., 2018; Barrada et al., 2019; Castro and Santos-Iglesias, 2016; Castro et al., 2020; Fernández del Río et al., 2019). This appears in the description of the study's inclusion criteria.

Second, as is highlighted in some reviews on the use of dating apps (e.g., Castro & Barrada, 2020), emerging adults are the most studied group and in which higher rates of use of these applications have been found. This idea already appeared in the previous version of the manuscript. However, following your recommendations, we have added an explanation of the differences in use and motivations for use, to highlight the relevance of focusing on this population (p. 3, lines 49-55):

*The motivations for using dating apps are determined by the users’ individual characteristics [1]. Sociodemographic variables (i.e., sex, age, and sexual orientation) are those with a higher relationship with the use of apps [9,10]. Specifically, past literature highlighted that men [6,10], and members of sexual minorities [6,10,11], present higher prevalence rates for the use of dating apps. Based on age, the most studied group and in which higher rates of app use is older youth, who tend to show a wide variety of motives to use it, seeking both entertainment and casual sex or romantic partner [2,4,10].*

Finally, from an evolutionary perspective, we understand the relevance of this population for mating. We start from the assumption that mating is a lifelong process with great implications for future life, as we already pointed out in the manuscript. However, in the study of mating, special attention has been paid to some specific stages of life, such as youth. In this stage, they are made decisions (e.g., looking for a partner with whom to have a committed romantic relationship and have children, or on the contrary to seek only casual sexual relationships, or to seek both types of relationships, or not to seek neither) that can influence the rest of people's lives and determine their future relationships (that is, partner, parenthood/motherhood) and behaviors (Buss & Schmitt, 2019). Following your recommendations, we have completed the paragraph in the Introduction that talks about the importance of mating (now, on p. 4, lines 71-83).

*Continuing with the influence of individual differences, the literature has paid particular attention to mating preferences and orientations. Mating is a lifelong process [20,21] with great implications for future life [22,23]. Traditionally, its relevance has been emphasized during emerging adulthood, when decisions are often made about relationships and offspring, events that have a considerable impact on peoples’ lives. [20,21]. Mating orientation, the individuals’ stated interest in committed relationships and/or in brief or uncommitted sexual relationships [24], has usually been measured through a single dimension with two opposite poles: short-term versus long-term [21]. Short-mating orientation is characterized by the search for casual sexual partners and relationships of low emotional commitment [21,24,25], and traditionally has been identified with unrestricted sociosexuality. Long-term mating orientation, on the other hand, is characterized by the desire for romantic relationships of commitment, with a strong emotional investment in the relationship and, generally, with sexual exclusivity [26].*

**Finally, the authors have adequately addressed reviewer concerns about generalisability. The authors include good discussion, particularly in relation to the increasing % of sexual minority participants. However, although this trend is applicable in Spain, the authors have not really addressed if this is generalised to other countries?**

Response: Thank you very much for your words. In the previous version of the manuscript, and in response to one of the reviewers, we tried to explain this increase in the prevalence of members of sexual minorities, and we indicated that our analysis included sexual orientation as covariate:

*First, our surveys are not just biased by sexual orientation (higher probability of participation for non-heterosexual people), but also that bias is growing. We cannot find any theoretically plausible explanation for this potential change of bias across time. Second, in fact in the population of university students (Spain, a single university) the presence of non-heterosexuality is increasing. This second alternative would imply that the large number of non-heterosexual participants is not a problem of representativeness of the samples. Further research is needed to clarify this point. In any case, in our regression analyses we included sexual orientation as covariate. In addition, to facilitate the analyses, we decided to group participants into heterosexuals and non-heterosexuals, thus losing the nuances related to the behavior of members of sexual minorities.*

This prevalence of sexual minorities is higher than that found in similar studies in other countries, both European (for example, Sumter & Vandenbosch (2019), in the Netherlands, had an 83.5% of heterosexuals and 16.5% of non-heterosexuals) as in the United States (for example, Alexopoulos et al. (2020), found 80.5% of heterosexuals and 19.5% of non-heterosexuals). Nevertheless, this prevalence is quite similar to research in other countries, like this by Tsoukas and March (2018) among Australians aged 18 to 69 (71% heterosexuals, 29% non-heterosexuals).

Besides that, one of the latest studies published on this topic is that of Rahman et al. (2020), who assessed the prevalence of women's and men's sexual orientation in 28 nations using data from 191,088 participants. These authors found that, in terms of sexual attraction (one of de components of sexual orientation), 82.6% of men and 66.2% of women were predominantly not attracted to the same sex, while 17.4% of men and 33.8% of women were moderately or predominantly attracted to the same sex. Considering data by country, with data grouped together for men and women, it can be seen that the proportion for Spain is 73% vs. 27%, similar to that of this study and very similar to that of other countries, such as Australia (74%-26%), Finland (71%-29%), Netherlands (70%-30%), or United States (73%-27%). Therefore, it seems that in terms of sexual attraction, the data from other countries are similar to ours. As for the trend, it seems that in recent years and especially in countries with more tolerant laws towards sexual minorities, such as Spain, there is an increase in the prevalence of people who claim to belong to them, a process parallel to the decrease of stigma and an improvement in the quality of life of these people (Pachankis et al., 2016).

Taking all of these into account, we have modified the Discussion section, briefly pointing out that the results obtained and the trend in our data may be similar to what is observed in other countries (now, in p. 16-17, lines 334-361):

*Concerning to sexual orientation, two aspects should be noted. First, the high proportion of participants from sexual minorities, more than 30% of the final sample. This could be considered as a lack of representativeness of our sample. We consider that an alternative interpretation is possible. This study shares with previous studies the same sampling approach and population (Spanish university students with the same age range and from the same university). We will show the time of data collection and the proportion of sexual minority participants: November 2018, 27.0% [14], December 2017, 22.5% [9], May 2016, 14.7% [38], April 2016, 12.7% [35], October 2013, 8.6% [39]. A clear trend is found. The proportion of sexual minority participants is steadily increasing in our samples.*

*We can imagine two options to explain this. First, our surveys are not just biased by sexual orientation (higher probability of participation for non-heterosexual people), but also that bias is growing. We cannot find any theoretically plausible explanation for this potential change of bias across time. Second, in fact in the population of university students (Spain, a single university) the presence of non-heterosexuality is increasing. This second alternative would imply that the large number of non-heterosexual participants is not a problem of representativeness of the samples.*

*This hypothesis may be supported by data on the prevalence of persons from sexual minorities found in other studies, which can be exemplified in that of Rahman et al. [40], who assessed the prevalence of women's and men's sexual orientation in 28 nations and found similar proportions to those of the present study, both in Spain (73% vs. 27%) and in other countries. There seems to be a trend toward greater self-identification as a member of sexual minorities, paralleling the decrease in stigma and the improvement in the quality of life of these people, especially in countries with more tolerant laws, as is the case in Spain [41]. However, further research is needed to clarify this point. And, in any case, in our regression analyses, we included sexual orientation as a covariate. In addition, to facilitate the analyses, we decided to group participants into heterosexuals and non-heterosexuals, thus losing the nuances related to the behavior of members of sexual minorities.*

**I also do not understand the statement (line 335): 'the problem of representativeness is more apparent that real'.**

Response: We have changed this sentence to (p. 17, lines 347-349):

*This second alternative would imply that the large number of non-heterosexual participants is not a problem of representativeness of the samples*

**References**

Alexopoulos, C., Timmermans, E., & McNallie, J. (2020). Swiping more, committing less: Unraveling the links among dating app use, dating app success, and intention to commit infidelity. *Computers & Human Behavior, 102,* 172-180. https://doi.org/10.1016/j.chb.2019.08.009

Barrada, J. R., & Castro, A. (2020). Tinder users: Sociodemographic, psychological, and psychosexual characteristics. *International Journal of Environmental Research and Public Health*, *17*(21), 8047. https://doi.org/10.3390/ijerph17218047

Barrada, J. R., Castro, A., Correa, A. B., & Ruiz-Gómez, P. (2018). The tridimensional structure of sociosexuality: Spanish validation of the Revised Sociosexual Orientation Inventory. *Journal of Sex & Marital Therapy*, *44*, 149–158. https://doi.org/10.1080/0092623X.2017.1335665

Barrada, J. R., Ruiz-Gómez, P., Correa, A. B., & Castro, A. (2019). Not all Online Sexual Activities are the same. *Frontiers in Psychology, 10,* 339. https:/doi.org/10.3389/fpsyg.2019.00339

Buss, D. M., & Schmitt, D. P. (2019). Mate preferences and their behavioral manifestations. *Annual Review of Psychology*, *70*, 77–110. https://doi.org/10.1146/annurev-psych-010418-103408

Castro, A., & Barrada, J. R. (2020). Dating apps and their sociodemographic and psychosocial correlates: A systematic review. *International Journal of Environmental Research and Public Health, 17,* 6500. https://doi.org/10.3390/ijerph17186500

Castro, A., Barrada, J. R., Ramos-Villagrasa, P. J., & Fernández del Río, E. (2020). Profiling dating app users: Sociodemographic and personality characteristics. *International Journal of Environmental Research and Public Health, 17,* 3652. https://doi.org/10.3390/ijerph17103653

Castro, A., & Santos-Iglesias, P. (2016). Sexual behavior and sexual risks among Spanish university students: A descriptive study of gender and sexual orientation. *Sexuality Research and Social Policy, 13,* 84-94. https://doi.org/10.1007/s13178-015-0210-0

Fernández del Río, E., Ramos-Villagrasa, P. J., Castro, A., & Barrada, J. R. (2019). Sociosexuality and bright and dark personality: The prediction of behavior, attitude, and desire to engage in casual sex. *International Journal of Environmental Research and Public Health, 16,* 2731. https://doi.org/10.3390/ijerph17186500

Jackson, J. J., & Kirkpatrick, L. A. (2007). The structure and measurement of human mating strategies: Toward a multidimensional model of sociosexuality. *Evolution and Human Behavior*, *28*, 382–391. https://doi.org/10.1016/j.evolhumbehav.2007.04.005

Pachankis, J. E., Haztenbuehler, M. L., Mirandola, M., Weatherburn, P., Berg, R. C., Marcus, U., et al. (2016). The geography of sexual orientation: Structural stigma and sexual attraction, behavior, and identity among men who have sex with men across 38 European countries. *Archives of Sexual Behavior, 46,* 1491-1502. https://doi.org/10.1007/s10508-016-0819-y

Penke, L., & Asendorpf, J. B. (2008). Beyond global sociosexual orientations: A more differentiated look at sociosexuality and its effects on courtship and romantic relationships. *Journal of Personality and Social Psychology*, *95*(5), 1113–1135. https://doi.org/10.1037/0022-3514.95.5.1113

Rahman, Q., Xu, Y., Lippa, R. A., & Vasey, P. L. (2020). Prevalence of sexual orientation across 28 nations and its association with gender equality, economic development, and individualism. *Archives of Sexual Behavior, 49,* 595-606. https://doi.org/10.1007/s10508-019-01590-0

Simpson, J. A., & Gangestad, S. W. (1991). Individual differences in sociosexuality: Evidence for convergent and discriminant validity. *Journal of Personality and Social Psychology*, *60*(6), 870–883. https://doi.org/10.1037/0022-3514.60.6.870

Sumter, S. R., & Vandenbosch, L. (2019). Dating gona mobile: Demographic and personality-based correlates of using smartphone-based dating applications among emerging adults. *New Media & Society, 21,* 655-673. https://doi.org/10.1177/1461444818804773

Tsoukas, A., & March, E. (2018). Predicting short- and long-term mating orientations: The role of sex and the Dark Tetrad. *Journal of Sex Research, 55,* 1206-1218. https://doi.org/10.1080/00224499.2017.1420750
